# Targeted Photodynamic Therapy Using Alloyed Nanoparticle-Conjugated 5-Aminolevulinic Acid for Breast Cancer

**DOI:** 10.3390/pharmaceutics13091375

**Published:** 2021-08-31

**Authors:** Hanieh Montaseri, Cherie Ann Kruger, Heidi Abrahamse

**Affiliations:** Laser Research Centre, Faculty of Health Sciences, University of Johannesburg, P.O. Box 17011, Doornfontein 2028, South Africa; haniehm@uj.ac.za (H.M.); cherier@uj.ac.za (C.A.K.)

**Keywords:** alloyed bimetallic nanoparticles, 5-aminolevulinic acid, targeted photodynamic therapy, MCF-7 breast cancer treatment

## Abstract

Photodynamic therapy (PDT) has been investigated as an effective, non-invasive, and alternative tumor-ablative therapy that uses photosensitizers (PSs) and safe irradiation light in the presence of oxygen to generate reactive oxygen species (ROS) to kill malignant cancer cells. However, the off-target activation of the PSs can hinder effective PDT. Therefore, an advanced drug delivery system is required to selectively deliver the PS to the therapeutic region only and reduce off-target side effects in cancer treatment. The integration of laser-initiated PDT with nanotechnology has provided new opportunities in cancer therapy. In this study, plasmonic bimetallic nanoparticles (NPs) were prepared for the targeted PDT (TPDT) of in vitro cultured MCF-7 breast cancer cells. The NPs were functionalized with PEG through Au–thiol linkage to enhance their biocompatibility and subsequently attached to the PS precursor 5-aminolevulinic acid via electrostatic interactions. In order to enhance specific targeting, anti-HER-2 antibodies (Ab) were decorated onto the surface of the nanoconjugate (NC) to fabricate a 5-ALA/Au–Ag-PEG-Ab NC. In vitro studies showed that the synthesized NC can enter MCF-7 cells and localize in the cytoplasm to metabolize 5-ALA to protoporphyrin IX (PpIX). Upon light irradiation, PpIX can efficiently produce ROS for the PDT treatment of MCF-7. Cellular viability studies showed a decrease from 49.8% ± 5.6 ** to 13.8% ± 2.0 *** for free 5-ALA versus the NC, respectively, under equivalent concentrations of the PS (0.5 mM, IC50). These results suggest that the active targeted NC platform has an improved PDT effect on MCF-7 breast cancer cells.

## 1. Introduction

Breast cancer is the most common malignancy in women and affects approximately one-eighth of women worldwide [1]. Although some promising therapeutic modalities, such as chemotherapy, radiotherapy, and surgery, have been developed, the major setback is still associated with traditional techniques, whereby they affect healthy cells and induce major side effects [2]. Laser-induced photodynamic therapy (PDT) together with photosensitizer (PS) delivery has emerged to overcome the limitations of conventional therapies and obliterate cancer cells.

PDT is a minimally invasive cancer therapeutic modality that is based on the administration of a photosensitizer (PS) to cancer tissues followed by irradiation, at an appropriate wavelength of light, to excite the PSs to produce reactive oxygen species (ROS) to obliterate neoplastic tissue [3]. Compared with other modalities, PDT provides minimal side effects, low morbidity, and good tolerance [3].

Among various PSs, 5-aminolevulinic acid (5-ALA) is an FDA-approved PS precursor that has been extensively utilized in PDT in cancer therapy and is considered to be a protoporphyrin IX (PpIX) precursor with low dark toxicity and rapid clearance from the body (24–48 h) [4,5]. 5-ALA is internalized by cancer cells through peptide transporters 1 and 2 and converted to protoporphyrin IX (PpIX) inside the mitochondrion [6]. PpIX is a photosensitive fluorescent molecule that degrades at different rates in cancer and normal cells [7]. The difference in PpIX accumulation in cancer and normal cells is driven by its active conversion through the heme biosynthetic pathway and ferrochelatase (FECH) [6,8]. Inactivation and downregulation of FECH play pivotal roles in selective accumulation of PpIX in cancer cells [9], while in normal cells FECH is positively regulated at transcriptional and translational levels [8]. Furthermore, it has been reported that iron chelators can bind Fe^2+^ ions, so there is less Fe^2+^ available for the FECH-catalyzed deactivation of the PpIX, leading to an enhancement in the efficacy of ALA-PDT [10,11].

PpIX has four different absorption bands (located at 506 nm, 542 nm, 575 nm, and 630 nm) [12]. The shorter wavelengths of light have a limited penetration depth in all tissues compared with the longer wavelengths, so 630 nm (the longer wavelength of PpIX) is utilized in PDT treatments to excite this PS [12]. Furthermore, the hydrophilic properties of 5-ALA often restrain its internalization and specific lipophilic cell membrane targeting abilities [12]. To overcome this obstacle, nanocarriers offer high tumor targeting efficiency and subcellular uptake for 5-ALA via passive or active targeting [13]. The permeability and retention effect (EPR) plays a pivotal role in passive targeting [13], while the binding of specific targeting ligands, such as antibodies (Abs) or folic acid, onto the surface of PS-loaded nanocarrier systems is of great importance to active targeting requirements [13]. However, active targeting can be utilized as a complementary approach to EPR-passive targeting to enhance the NCs’ accumulation and retention [14]. Additionally, EPR-mediated tumor accumulation is associated with long-circulation NCs with a hydrodynamic diameter size (in the size range of 20–200 nm) greater than the renal clearance threshold. These NCs can extravasate from leaky tumor vessels and that accumulate inside the interstitial space [13,14].

Several studies have noted that nanocarriers are capable of overcoming the externalizing activity of PpIX from tumor cells [15]. Among the various nanocarriers of PDT for MCF-7 breast cancer cells [16,17,18,19,20,21,22], plasmonic NPs have gained considerable research interest [23]. They are versatile nanocarriers with unique physical and chemical properties enabling them to carry various therapeutic payloads to specific tissue sites. They can be easily synthesized in monodispersed sizes that are inert and non-toxic in nature. Furthermore, they are easily functionalized with drugs and targeting ligands [24]. More importantly, when plasmonic NPs are activated with laser light irradiation, they generate heat and so photothermal energy is produced that also enhances PDT outcomes [25]. Among the plasmonic NPs, alloyed bimetallic NPs, such as gold and silver, are suitable for therapeutic applications and imaging due to the combination of plasmonic features of gold and silver [26,27].

In this work, 5-ALA/AuAg-PEG-Ab nanoconjugates (NCs) were synthesized via electrostatic interactions. The NCs are comprised of a 5-ALA PS precursor and a targeting ligand anti-HER-2 antibody (Ab) for the PDT of in vitro cultured MCF-7 breast cancer cells. The alloyed bimetallic Au–Ag NPs were further coated with SH-PEG-NH_2_ through Au–S interactions. Photosensitive 5-ALA was then electrostatically conjugated to the PEGylated NPs. The anti-HER-2 Ab was used in order to try and enhance the PS cellular uptake of the NCs for improved PDT treatment outcomes. In order to confirm the efficacy of the NCs for cancer therapy, the physiochemical properties of the NCs, including morphology, size, zeta potential, and stability, were evaluated. Furthermore, the subcellular uptake, cellular viability, and PDT therapeutic effect of the NCs were investigated within MCF-7 cells and showed promising results. Ultimately, the results suggest that modification of the ALA structure could have implications for the use of NCs with active moieties in photodynamic therapy of breast cancer cells.

## 2. Materials and Methods

### 2.1. Materials

Gold (III) chloride trihydrate (HAuCl_4_.3H_2_O, ≥99.9% trace metals basis), tri-sodium citrate (for molecular biology, ≥99%), tannic acid (ACS reagent), silver nitrate (AgNO_3_, ACS reagent, ≥99.0%), SH-PEG2k-NH_2_ (HCl salt, average M_n_: 2000), 5-aminolevulinic acid hydrochloride (5-ALA∙HCl, ≥98%), and phosphate-buffered saline (PBS, 10× concentrate, BioPerformance Certified, suitable for cell culture) were supplied by Sigma Aldrich. Monoclonal anti-HER-2 Ab was purchased from Biocom Africa (Pty) Ltd., Johannesburg, South Africa. All chemicals and reagents used in the study were of analytical grade. Millipore water with a resistance of 18.25 MΩ cm was used to prepare aqueous solutions. All glassware was rinsed with aqua regia solution before synthesis of the NPs.

### 2.2. Synthesis of Bimetallic Alloyed Au–Ag NPs

To synthesize bimetallic alloyed citrate AuAg NPs, 1 mL of 1% HAuCl_4_.3H_2_O and 1 mL of AgNO_3_ were mixed in a three-neck flask with 78 mL of Millipore water in a reflux system. A solution containing 4 mL of 1% tri-sodium citrate, 0.5 mL of tannic acid, and 15.5 mL of Millipore water was then added to the flask and stirred for 30 min at ~60 °C. The synthesized NPs were kept at 4 °C for further experiments.

### 2.3. Functionalization of the Au–Ag NPs with PEG, 5-ALA, and Ab

The negatively charged surface of alloyed citrate Au–Ag NPs was changed to a positively charged surface by mixing the NPs with SH-PEG2k-NH_2_, which was hydrolyzed by KOH. Briefly, 5 mg/mL of SH-PEG2k-NH_2_ and 10 mg of KOH were dissolved in PBS to prepare a ligand exchange solution. One milliliter (1 mL) of citrate-capped Au–Ag alloyed NPs was then added to 1 mL of ligand exchange solution, followed by stirring for a few minutes to encourage Au–S linkage. The excess PEG was removed by centrifugation and the precipitate was dispersed in PBS.

In order to attach negatively charged 5-ALA via electrostatic interaction to the Au–Ag-PEG NPs, 1 mL of 0.3 M 5-ALA was mixed with 1 mL of the NPs for 2 h. The mixture was centrifuged, and the precipitate was redispersed in PBS to fabricate 5-ALA/Au–Ag-PEG NPs.

For the specifically targeted PDT of MCF-7 cells, anti-HER-2 Ab was conjugated with NPs. A total of 6 µg/mL of anti HER-2 Ab was mixed with 5-ALA/Au–Ag-PEG NCs and incubated for 2 h at 4 °C in the dark, followed by centrifugation and dispersion of the precipitate in PBS. The antibodies were bound to the surface of the PEG-coated Au–Ag NPs by electrostatic physisorption interactions [28,29,30,31]. The final 5-ALA/Au–Ag-PEG-Ab NCs were kept at 4 °C in the dark for further experiments. The conjugation of 5-ALA and Ab to the PEGylated NPs was confirmed through various characterization techniques.

### 2.4. Characterization of NCs

The UV/Vis spectra of the NCs were recorded using a Jenway GenovaNano Plus Life Science Spectrophotometer, Cole-Parmer Ltd, Stone, Staffordshire, UK. TEM analysis was performed to confirm the size and morphology of the synthesized NPs using a JEOL, software JEM-2100, a 200 kV accelerating voltage, and a CCD camera (Gatan; Inc. Coronado Lane, Japan). The estimated particle size distribution of the NPs was determined using ImageJ software (http://imagej.nih.gov/ij/ (accessed on 22 June 2021)), Java 1.8.0_112, U.S. National Institute of Health (NIH), Bethesda, MD, USA). Dynamic light scattering (DLS) and electrophoretic light scattering (ELS) or zeta potential analysis of the NCs were carried out using Malvern Zetasizer Nano ZS (Malvern Instruments, Zetasizer software, 7.03, Malvern, UK) with a 4 mW He-Ne laser of 633 nm wavelength in order to investigate the charge and size of the NCs. FT-IR analysis was performed using the Spectrum 100, FT-IR Spectrometer (PerkinElmer Spectrum, Version 10.03.02, Bucks, UK) in the range of 400–4000 cm^−1^ with 25 scans.

### 2.5. Cell Culture and Preparation of Cell Culture Plates

MCF-7 breast cancer cells (American Type Cell Culture, ATCC, Manassas, VA, USA, ATCC HTB-22) were seeded as adherent monolayers in culture media containing Dulbecco’s Modified Eagle’s Medium (DMEM, Sigma-Aldrich, D5796), 10% (*v*/*v*) fetal bovine serum (FBS, Thermo Fisher Scientific, 10499-044), 1% (*v*/*v*) penicillin/streptomycin (Sigma-Aldrich, P4333), and 1% (*v*/*v*) amphotericin-β (Sigma-Aldrich, A2942) at 37 °C in a humidified atmosphere of 5% CO_2_.

Once MCF-7 monolayer cells were achieved (at approximately 85% confluency), they were detached with Tryple ™Select (Gibco Invitrogen, 12563-029). The cell suspensions were then sub-cultured into 3.4 cm diameter cell culture plates at a seeding ratio of 1.0 × 10^5^ cells/mL in 3 mL of complete cell growth medium and incubated for 4 h to allow for cellular attachment.

### 2.6. PDT Laser Parameters and PS Addition

After 4 h of incubation, the culture plates were divided into various control and experimental groups, received ranging concentrations of either the 5-ALA PS precursor or the NC in serum-free media, and were then incubated for 20 h. Following incubation, the various control and experimental groups that required PDT treatment received laser light irradiation in PBS.

Drug transporters, such as ATP binding cassette (ABC), and solute carrier (SLC) protein transporters play an important role in drug-metabolizing enzymes and the pharmacological effects of drugs. In ALA-induced PpIX, peptide transporter 1 (PEPT1; SLC15A1) and PEPT2 (SLC15A2) are involved in the cellular uptake of 5-ALA [10]. While 5-ALA influx could be mediated by PEPT1 and PEPT2, upregulation of ABCG2 could lead to PpIX efflux and enable cancer cells to survive after ALA-PDT [10]. ABCG2 is overexpressed in many cancer cells and functions to transport porphyrins in an ATP-dependent manner. It also protects cells against phototoxicity by mediating the efflux of porphyrins from them [32]. It was reported that an increase in PpIX efflux and activation of ABCG2 [33] in cancer cells can occur in the presence of serum [32,34,35].

Clinical PSs such as PpIX are transported out of the cells by the action of ABCG2. However, co-administration of ALA with imatinib mesylate [36], gefitinib, or other ABCG2 transport inhibitors [37] could enhance the efficacy of clinical PDT [32].

Additionally, cellular efflux pumps and enzymatic conversion of PpIX to photochemically inactive heme could be affected by glycolysis inhibitors, resulting in lower accumulation of PpIX in cancer cells [38]. Therefore, serum-free media was used for PDT of MCF-7 in this study to decrease PpIX efflux from cancer cells.

All laser light irradiations were performed using a 636 nm diode laser (supplied by the National Laser Centre, Pretoria, South Africa) at a fluence of 5 J/cm^2^ (light intensity: approximately 8 mW/cm^2^) over 10 min. The culture media of all plates were then replaced with fresh complete media and incubated for an additional 24 h before various biochemical assays were performed. All experiments were carried out at room temperature in the dark. Irradiation time was calculated as follows:

(1) mWcm2=mW×4π×3.42

(2) Wcm2=mWcm21000

(3) Time (s)=Jcm2Wcm2

where mW is the laser output, mW/cm^2^ is the light intensity, and J/cm^2^ is the light fluency. Moreover, πD^2^/4 is the area of a circle for 3.4 cm diameter (D) culture plates.

### 2.7. In Vitro Subcellular Localization of the PS and NCs

A total of 1.0 × 10^5^ cells/mL of MCF-7 cells were seeded into 3.4 cm diameter culture plates containing sterile cover slips and incubated for 4 h at 37 °C to allow for cellular attachment. After 4 h incubation, the media were replaced with serum-free media containing the PS or the NC. The plates were then incubated at 37 °C for an additional 20 h in the dark. Following incubation, the cells were rinsed with PBS and 4% paraformaldehyde (Sigma Aldrich, P6148) was added to fix the cells onto the coverslips, followed by 15 min of incubation. The cells were then permeabilized with 0.5% Triton X-100 (Sigma Aldrich, T9284) in 1X PBS for 15 min. Then, the cells were counter stained with 300 nM of 4′,6-diamidino-2-phenylindole (DAPI, Invitrogen™, D1306) for 5 min and the coverslips were inverted onto a glass slide with a drop of fluoromount aqueous mounting medium (Sigma Aldrich, F4680). The slides were sealed using a transparent nail polish and observed on the Carl Zeiss Axio Observer Z1, Zen 3.1 (blue edition), Zeiss, Johannesburg, South Africa (a live imaging microscope). All preparations were carried out at room temperature in the dark.

### 2.8. In Vitro Photodynamic Therapy of NCs

#### 2.8.1. Morphology

The cellular morphological changes in the MCF-7 cells were assessed 24 h post irradiation using a light inverted microscope at 100× magnification with a digital camera (Olympus C5060-ADUS, cellSens Entry, XV Image Processing, Wirsam Scientific, Johannesburg, South Africa).

#### 2.8.2. ATP Cell Viability Assay

The ATP viability assay was performed to investigate the viability of MCF-7 cells within various control and experimental groups, which included 5-ALA/Au–Ag-PEG, 5-ALA/Au–Ag-PEG-Ab NC, and free 5-ALA, with and without laser light irradiation. In a typical procedure, 1.0 × 10^5^ cells/mL were seeded in 3.4 cm diameter culture plates for 4 h. Then, different concentrations of the various control and experimental groups of PS or NCs were added to the cells in serum-free media. They were incubated at 37 °C for an additional 20 h. Following incubation, the media of culture plates that required laser light irradiation were replaced with PBS and they were irradiated. The culture media of all plates were then replaced with fresh complete media, followed by a 24 h incubation.

Post 24 h incubation, the adenosine triphosphate (ATP) luminescence for cell viability was measured using the CellTiter-Glo^®^ Luminescent cell viability assay (Promega, G7570, Madison, WI, USA). Briefly, 100 µL of cell suspension was mixed with an equivalent volume of the ATP CellTiter-Glo^®^ reagent in an opaque-walled 96-well plate and then lysed for 2 min followed by 10 min of incubation at room temperature in the dark. The luminescent signal of the cells was measured with a VICTOR Nivo^®^ multimode plate reader (PerkinElmer, HH35940080 EN, VICTOR Nivo software 2.5, Waltham, MA, USA) and the signal was detected in relative light units (RLUs). The cell viability was calculated by subtracting background luminescence values from experimental readings in order to obtain true values.

#### 2.8.3. Flow Cytometry for Apoptosis and Necrosis

In order to investigate early or late apoptotic and necrotic phases of cells’ death, the Annexin V-FITC/PI cell death detection kit (BD Scientific: BD/556570) was used following the manufacturer’s instructions and the results were read using a BD Accuri™ C6 flow cytometer. MCF-7 cells were seeded in small plates and incubated for 4 h. The cells were then treated with free 5-ALA, 5-ALA/Au–Ag-PEG, and 5-ALA/Au–Ag-PEG-Ab NCs and incubated for an additional 20 h. Control and experimental groups that required PDT treatment received laser light irradiation. All culture plates were incubated for an additional 24 h, after which they were detached and stained with Annexin V-FITC/PI.

### 2.9. Statistical Analysis

Biochemical assays were carried out in duplicate for six independent experiments. Spectrophotometry measurements were performed against a blank sample based on the media of each sample. Student’s *t*-test was used to compare two groups and one-way ANOVA was used to compare all groups together, in order to determine the significance of the difference between the various control and experimental groups. Values in the 95% confidence interval (*p* < 0.05 *, *p* < 0.01 **, or *p* < 0.001 ***) were accepted as statistically significant and the standard error was defined by dispersion bars.

## 3. Results and Discussion

### 3.1. Synthesis of the NCs

**Scheme 1:**
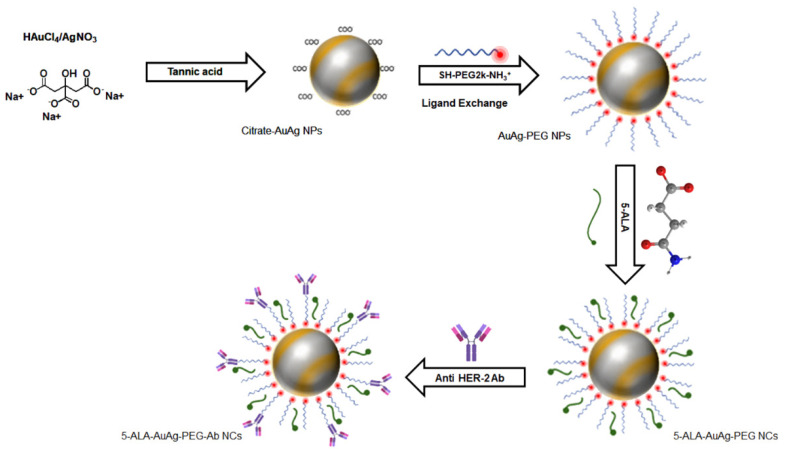
The main steps of AuAg NP synthesis followed by surface PEGylation and conjugation with 5-ALA and anti HER-2 Ab.

Au–Ag NPs with the approximate size of 16.4 ± 3.9 nm with long-term stability were obtained. The high stability of the synthesized bimetallic citrate Au–Ag NPs was due to the presence of sodium citrate, which induces an abundance of negative charges, creating repulsions between NPs and so no aggregation was observed [39]. This finding was confirmed by UV-Visible spectroscopy and the reported polydispersity indexes (PDIs). The synthesis protocol of citrate Au–Ag NPs, followed by functionalization with PEG, 5-ALA, and anti HER-2 Ab, is illustrated in Scheme 1. Since citrate molecules may induce cytotoxicity and prevent drug loading, the synthesized NPs were functionalized with SH-PEG-NH_2_ through Au–thiol bonds to purge the citrate molecules from the NC. Additionally, various concentrations of SH-PEG-NH_2_ (0.5–10 mg/mL) were mixed with citrate Au–Ag NPs in order to allow for the optimum feeding ratio of the SH-PEG-NH_2_ with the highest stability to be chosen. PEGylation of NPs not only improves their biocompatibility under physiological conditions and blood circulation lifetime, but also reduces their uptake by reticuloendothelial systems and removal by the liver or spleen [40].

If required, the concentration of Au–Ag NPs can be measured by ICP-MS followed by plotting a calibration curve in order to calculate the number of PEG, 5-ALA, and Abs attached onto the surface of the NPs.

### 3.2. Molecular Characterizations of the NCs

**Scheme 2:**
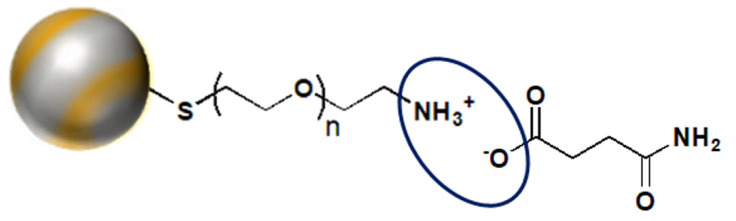
Electrostatic interactions between AuAg-S-PEG-NH_2_ and 5-ALA.

Citrate Au–Ag NPs were prepared by reduction of the metals using sodium citrate and tannic acid to induce the growth of seeds for NP synthesis. The ligand exchange reaction was then carried out to functionalize the surface of NPs with SH-PEG-NH_2_. PEGylation of the nanomaterials can lead to sufficient stability in aqueous media as well as induce strong resistance towards unwanted biomolecule binding [41]. Since the Au–Ag-S-PEG-NH_2_ NPs were positively charged, they could be conjugated to 5-ALA with anionic properties. 5-ALA is a zwitterion that has COO^−^ and -NH_3_^+^ groups and so the pH of the solution determines the charge it carries. If a solution is acidic, it remains positively charged due to the presence of the -NH_3_^+^ and if the solution is basic, it remains negatively charged due to the presence of COO^−^ groups [17]. Within physiological pH (7.2–7.4) solutions it also remains negatively charged [42]; hence, it can bind to positively charged Au–Ag-PEG-NH_2_ NPs through electrostatic interactions [43] (Scheme 2). Ultimately, the final positively charged 5-ALA-Au–Ag-PEG NCs could interact with the negatively charged segments of anti HER-2 Abs to form 5-ALA/Au–Ag-PEG-Ab NCs.

#### 3.2.1. UV–Visible Spectroscopy

**Figure 1:**
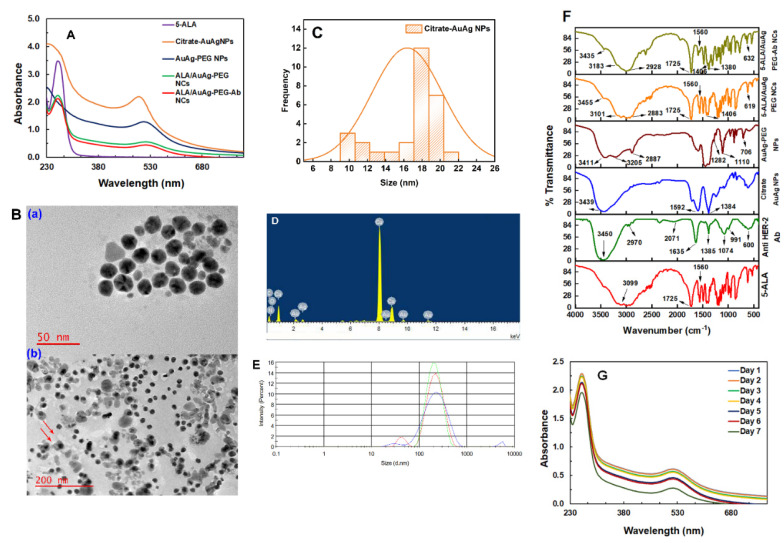
Characterization of the NCs. (**A**) UV-Visible spectra of 5-ALA, citrate Au–Ag NPs, 5-ALA/Au–Ag-PEG, and 5-ALA/Au–Ag-PEG-Ab NCs, (**B**) TEM images of (**a**) citrate Au–Ag NPs and (**b**) 5-ALA/Au–Ag-PEG-Ab NCs, (**C**) Particle size distribution of citrate Au–Ag NPs, (**D**) EDS spectra of 5-ALA/Au–Ag-PEG-Ab NCs, (**E**) Hydrodynamic size of 5-ALA/Au–Ag-PEG-Ab NCs by DLS, (**F**) FT-IR spectra of 5-ALA, anti HER-2 Ab, citrate Au–Ag NPs, Au–Ag-PEG NPs, 5-ALA/Au–Ag-PEG NCs, and 5-ALA/Au–Ag-PEG-Ab NCs, (**G**) photostability of 5-ALA/Au–Ag-PEG-Ab NCs in PBS over 7 days.

In order to synthesize alloyed bimetallic Au–Ag NPs, the precursor solutions of Au (HAuCl_4_.3H_2_O) and Ag (AgNO_3_) were mixed in the same reaction mixture to simultaneously form alloyed NPs [44]. A single localized surface plasmon resonance (LSPR) peak was observed for alloyed Au–Ag NPs at around 500 nm. Figure 1A shows the absorption spectra of 5-ALA, citrate Au–Ag NPs, PEGylated Au–Ag NPs, and conjugated NPs with 5-ALA and anti HER-2 Ab. The amine groups of SH-PEG-NH_2_ on the surface of the NPs could provide steric hindrance to prevent aggregation of the NPs [45,46]. The 5-ALA in PBS provided an absorbance peak at 261 nm, which was evident after conjugation to the PEGylated NPs (5-ALA/Au–Ag-PEG NCs). Regarding the conjugation of Ab, they generally possess a distinct absorption peak at 280 nm due to the presence of aromatic amino acid [47]. However, since 5-ALA has a strong absorption peak at 261 nm, this peak probably overlapped with the anti HER-2 Ab at 280 nm and so was not found to be clearly visible in Figure 1A. The presence of Ab in the final NC was confirmed by noting its higher cellular uptake, when compared with 5-ALA/AuAg-PEG NCs alone, using flow cytometry and ATP biochemical assays, which reported lowered cellular viabilities post-PDT when the final active targeted NCs were utilized. Similar absorption spectra were observed for the Au–Ag-PEG NPs and both NCs, indicating that functionalization did not affect the optical properties of the Au–Ag NPs or PS. Furthermore, the low PDI values and absorption spectra confirmed that no aggregation occurred during the conjugation [48]. It can be clearly observed that the localized surface plasmon resonance absorption (LSPR) of the PEGylated NPs and NCs is red-shifted. The coating of the surface, surrounding media, particle diameter, and particle shape have a significant impact on the LSPR absorption peaks of the NPs [49]. Moreover, an increase in the refractive index of the dielectric environment surrounding the plasmonic NPs upon PEG coating may also cause a red shift [50,51]. Additionally, it was noted that the binding of antibodies on the surface of the plasmonic NPs can also change the local dielectric environment of the NPs, resulting in a LSPR absorption peak shift [49,52].

#### 3.2.2. Morphological and Energy Dispersive Spectroscopy (EDS) Analysis

The TEM images of the NPs are illustrated in Figure 1B. Au–Ag NPs exhibited spherical NPs with good particle size monodispersity. Comparing the morphology of citrate Au–Ag NPs (Figure 1B(a)) and 5-ALA/Au–Ag-PEG-Ab NCs (Figure 1B(b)), the heterogeneous morphology of the particles clearly confirms the conjugation of the PEG, 5-ALA, and Ab to the Au–Ag NPs (shown with arrows). The structural feature of the active targeted NC reveals similarities with the plasmonic Au–Ag NPs with a well-dispersed morphology. The estimated average particle size distribution of the citrate Au–Ag NPs (Figure 1C) was found to be 16.4 ± 3.9 nm. Additionally, the hydrodynamic size distribution via dynamic light scattering further confirmed the attachment of the PEG, 5-ALA, and Ab onto the surface of Au–Ag NPs. EDS analysis was performed to confirm the presence of the metal components with the final NC, which reflected the presence of the Au and Ag (Figure 1D).

#### 3.2.3. Dynamic Light Scattering (DLS) and Zeta Potential (ZP)

DLS can be applied to investigate the size distribution of NPs. The electrokinetic potential or ZP was explored to confirm the colloidal stability of NPs in solution. Table 1 summarizes the hydrodynamic size, zeta potential, and polydispersity index (PDI) of the NPs and NCs. The average hydrodynamic size and zeta potential of the citrate Au–Ag NPs were 33.8 nm and −21 mV, respectively. After conjugation of the NPs with PEG, the size increased to 154.0 nm due to the large hydrodynamic volume of PEG, while the zeta potential decreased to 7.5 mV owing to the replacement of citrate with SH-PEH-NH_2_. Moreover, the polarity of the NPs was converted to a positive charge, indicating successful encapsulation of PEG molecules onto the surface of the NPs. The conjugation of 5-ALA to the PEGylated NP surface was confirmed by a further decrease in the zeta potential of the NPs to 5.5 mV [53]. Ultimately, after Ab loading, the hydrodynamic size and zeta potential were found to be 185.6 nm (Figure 1E) and 5.8 mV (Table 1), respectively. The smaller size of the 5-ALA/Au–Ag-PEG-Ab NCs, compared with the 5-ALA/Au–Ag-PEG NCs (192.4 nm), was probably due to the denser network arising from stronger electrostatic interactions between the 5-ALA/Au–Ag-PEG NCs and the Ab [54]. Additionally, the PDI below 0.4 for all NPs and NCs indicates an excellent distribution [55].

#### 3.2.4. FT-IR Analysis

The variation in the FT-IR spectra for the NPs and NCs is illustrated in Figure 1F. The FT-IR spectra of citrate-capped Au–Ag NPs showed two distinct absorption bands at 1592 and 1384 cm^−1^ arising from asymmetric and symmetric stretching vibrations of the sodium citrate COO^−^ groups. These peaks were found to be reduced after NP PEGylation, revealing the presence of PEG on the surface of Au–Ag NPs. The C-O-C stretching bands of HS-PEG-NH_2_ can be observed at 1280 and 1110 cm^−1^ in the spectra of Au–Ag-PEG NPs. A strong signal of C-H stretching vibrations from HS-PEG-NH_2_ was found at 2887, 2883, and 2928 cm^−1^ in Au–Ag-PEG NPs, 5-ALA/Au–Ag-PEG NCs, and 5-ALA/Au–Ag-PEG-Ab NCs, respectively. The primary amine group of the PEG is also represented with a characteristic band in the spectra of all PEGylated compounds at 3100–3200 cm^−1^. Moreover, the peak at 400–750 cm^−1^ can be related to the bonding of Au–Ag NPs with sulfur (Au–Ag-S) [39]. The 1725 cm^−1^ band can be associated with the stretching absorption of the C=O groups, which are present in the spectra of all NCs, confirming the attachment of 5-ALA. The characteristic peak at around 3400 cm^−1^ is due to the stretching of the O–H group from the intermolecular and intramolecular hydrogen bonds [56]. As can be seen in the spectra of 5-ALA/Au–Ag-PEG NCs and 5-ALA/Au–Ag-PEG-Ab NCs, two new peaks at 1560 and 1406 cm^−1^ were formed, which can be attributed to the N-H bending and stretching vibrations of NH_3_^+^, respectively [57], owing to the electrostatic interactions between amine groups of Au–Ag-S-PEG-NH_2_ and carboxyl groups of 5-ALA. The IR spectra of 5-ALA/Au–Ag-PEG-Ab NCs show peaks at 1385 cm^−1^ related to amide II that are similar to the peaks related to the anti HER-2 Ab [58].

#### 3.2.5. Photostability of 5-ALA/Au–Ag-PEG Ab NCs

The photostability of the NCs was evaluated over 7 days prior to laser light irradiation in order to monitor any photostability absorption changes in Au–Ag NPs and 5-ALA bands. As can be seen in Figure 1G, the absorption band of 5-ALA at 261 nm and the LSPR of the 5-ALA-Au–Ag-PEG-Ab NCs at around 516 nm showed a negligible decrease over 6 days. The decrease in the LSPR of the NCs is due to a decrease in the electron density in the NCs [59,60]. Despite the decline in the LSPR band of the NCs, no shift was noted over 7 days, which confirmed that the NCs stayed stable and monodispersed in PBS.

Despite the photostability of the NCs, previous studies have reported that 5-ALA is unstable in PBS at physiological pH, leading to a breakdown product that absorbs photons at 278 nm [61]. This breakdown decreases the availability of 5-ALA to convert to PpIX over prolonged storage, which will affect PDT treatment outcomes [61]. Although no breakdown band was seen at 278 nm over the 7-day photostability study, free 5-ALA solution and the NCs were always freshly prepared after 7 days and used throughout the study.

As mentioned above, 5-ALA was electrostatically attached onto the surface of the NPs. The NCs were stable in a physiological environment and any pH change can break these electrostatic interactions. Therefore, the acidic microenvironment of cancer cells can affect these interactions to release 5-ALA and accumulate it in cancer cells. Additionally, the presence of Abs can lead the NCs to bind to an antigen on the surface of the cancer cells only and enhance the 5-ALA’s internalization.

### 3.3. In Vitro Subcellular Uptake of the NCs

The biological half-life of PpIX in normal cells is 2–4 h, whereas in cancer cells it is reported to be 12–24 h [62]. Moreover, the accumulation of PpIX in the tumor cells is time dependent. Therefore, in a preliminary study, the time of uptake for free 5-ALA and the NCs after different incubation times was determined to be within 4 h and 20 h in order to achieve sufficient 5-ALA absorbance for effective PDT. However, the longest incubation time of 20 h was chosen in order to increase the 5-ALA accumulation and conversion to PpIX for maximum PDT phototoxicity in the MCF-7 cancer cells to be observed. These results are consistent with previous studies, where 5-ALA was incubated for varying incubation times within MCF-7 cells [12,63].

**Figure 2:**
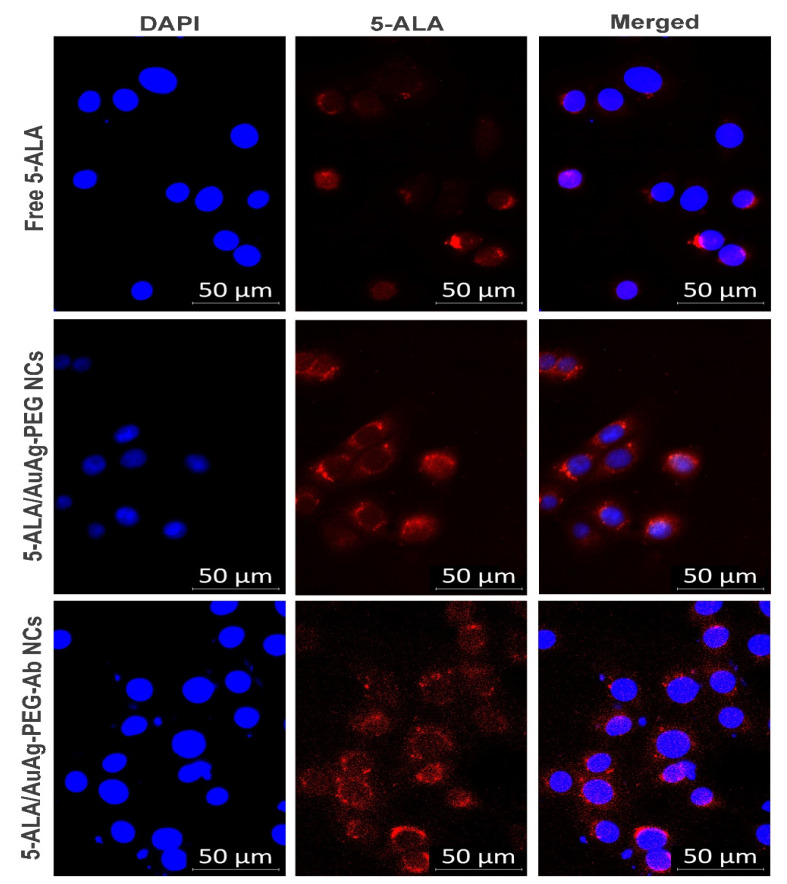
Subcellular localization of free 5-ALA, 5-ALA/Au–Ag-PEG, and 5-ALA/Au–Ag-PEG-Ab NCs under an equivalent concentration of 5-ALA after 20 h of incubation. The images in each row show DAPI-stained nuclei (blue), PpIX fluorescence (red), and their overlays, respectively.

In order to visually observe the cellular uptake of 5-ALA and conversion to PpIX, the red fluorescence of the PpIX in MCF-7 cells incubated under an equivalent concentration of 5-ALA for 20 h was carried out for free 5-ALA, 5-ALA/Au–Ag-PEG, and ALA/Au–Ag-PEG-Ab NCs and observed using a live imaging microscope. As shown in Figure 2, the cells treated with 5-ALA/Au–Ag-PEG-Ab NCs displayed stronger red fluorescence within their cytoplasm than those treated with free 5-ALA or 5-ALA/Au–Ag-PEG NCs. Furthermore, a higher uptake of the 5-ALA/Au–Ag-PEG-Ab NC within MCF-7 cells was observed. This finding confirmed active targeting, since the cells that were treated with the final NC generated more PpIX than the cells treated with free 5-ALA PS and 5-ALA/Au–Ag-PEG NCs, suggesting that the MCF-7 cells overexpressed anti HER-2 Ab.

### 3.4. In Vitro Photodynamic Therapy Studies

#### 3.4.1. Morphological Assessment

**Figure 3:**
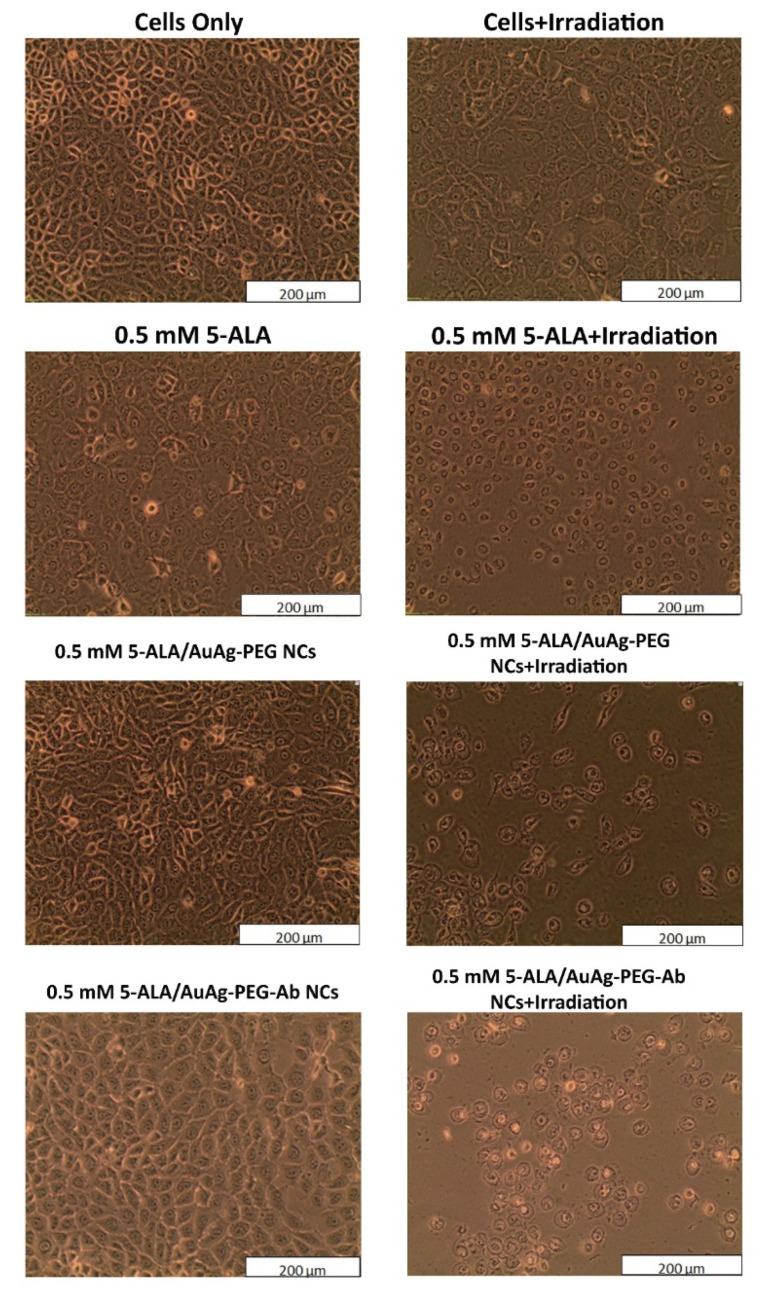
Light microscopy images for PDT-treated and non-treated MCF-7 cells with free 5-ALA, the NCs, and laser light irradiation under the IC50 concentration of 5-ALA at a wavelength of 636 nm and a fluency of 5 J/cm^2^.

Figure 3 shows morphological changes in MCF-7 cells under the IC50 concentration (0.5 mM) for free 5-ALA and 5-ALA/Au–Ag-PEG NCs as a control for passive targeting and 5-ALA/Au–Ag-PEG-Ab NCs as the final active targeted platform. Cellular morphology changes in treated MCF-7 cells with free 5-ALA and the NCs without laser light irradiation illustrated no significant damage when compared to cells only. These findings signify that the application of 5-ALA and the NCs alone induced no phototoxicity or dark toxicity. However, free-floating cells, detachment, and loss of cellular shape were observed for the cells treated with 5-ALA and the NCs in the presence of laser light irradiation. The most significant damage was noted for the PDT treatment of cells that received 5-ALA/Au–Ag-PEG-Ab NCs, whereby the cells appeared to be completely rounded up and detached. It can be concluded that NPs functionalized with an active targeting moiety can enhance 5-ALA PS cellular uptake, resulting in higher PDT efficacy.

#### 3.4.2. ATP Cell Viability Assay

**Figure 4:**
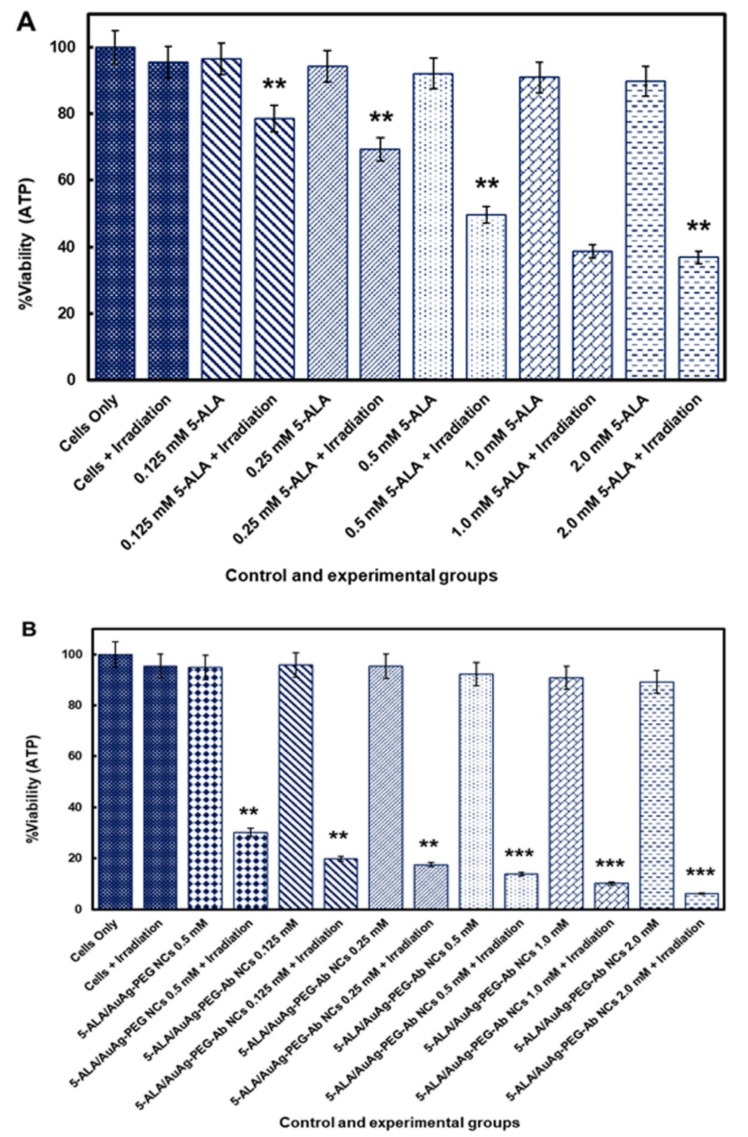
ATP cell viability assay of MCF-7 cancer cells treated with various concentrations of (**A**) free 5-ALA and (**B**) different concentrations of 5-ALA/Au–Ag-PEG NCs (control) and 5-ALA/Au–Ag-PEG-Ab NCs with and without light irradiation at 636 nm and a of fluency 5 J/cm^2^ (*p* < 0.05 *, *p* < 0.01 **, or *p* < 0.001 ***).

In order to evaluate the PDT therapeutic effects of 5-ALA and the NCs, MCF-7 cells were treated with different concentrations of free 5-ALA and the NCs under laser light irradiation at 636 nm with fluency of 5 J/cm^2^. As illustrated in Figure 4A, no significant dark toxicity was observed for the cells treated with free 5-ALA without laser light irradiation, even at the highest concentration of 2.0 mM. However, free 5-ALA plus laser light irradiation inhibited MCF-7 cell viability in a dose-dependent manner. In this regard, the lowest concentration of 5-ALA that could kill 50% of the cells (IC50) was found to be 0.5 mM.

Figure 4B compares the cell viability of MCF-7 cells that received 0.5 mM 5-ALA/Au–Ag-PEG NCs (as the passive PS loading NC control) and various concentrations of 5-ALA/Au–Ag-PEG-Ab NCs. Both NCs showed good cytocompatibility with negligible toxicity to MCF-7 cells without light irradiation. Yet, a lower cell viability of 30.2% ± 3.3 ** was observed for 0.5 mM 5-ALA/Au–Ag-PEG NPs plus irradiation when compared with 0.5 mM free 5-ALA (49.8% ± 5.6 **) plus irradiation. This confirmed that the NCs could passively deliver the PS to the cells via the EPR effect. In contrast, the lowest viability of 13.8% ± 2.0 *** was achieved within the final NC laser-light-treated cells, which were decorated with anti-HER-2 Ab in a dose-dependent manner. Therefore, the cell-killing efficacy was remarkably enhanced with anti-HER-2 Ab and the IC50 decreased significantly when compared with free 5-ALA, which was applied at the same concentration of 0.5 mM. From these results, it can be concluded that 5-ALA/Au–Ag-PEG-Ab NCs enhanced the targeted delivery of 5-ALA to the tumor cells and so resulted in more PpIX accumulation and PDT-induced cell death compared with free 5-ALA.

#### 3.4.3. Flow Cytometry Annexin V-FITC/PI Cell Death Pathway Detection Assay

In order to evaluate the capability of the actively targeted final NC to enhance PDT-induced cell death, flow cytometry was performed. Necrotic cell death is an undesirable PDT treatment outcome pathway, since it is characterized by loss of plasma membrane integrity and release of intracellular contents into the extracellular environment, resulting in adverse inflammatory and immunogenic responses [64]. Induced apoptotic forms of cell death post PDT treatment are far more favorable, since the cellular membrane’s integrity is maintained and so the release of harmful cellular waste to adjacent cells occurs with a far lower degree of inflammation [64].

**Figure 5:**
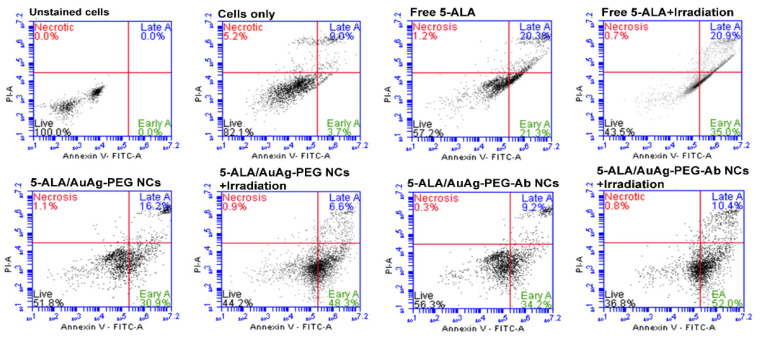
Flow cytometry analysis using the Annexin V-FITC/PI staining method on PDT-treated MCF-7 cells with Free 5-ALA, 5-ALA-Au–Ag-PEG, and 5-ALA-Au–Ag-PEG-Ab NCs under an equivalent concentration of 5-ALA.

The results of mechanisms of cell death by Annexin V-FITC/PI are shown in Figure 5. The percentage of apoptotic cells for untreated cells was 12.7% ± 0.1, while, in sharp contrast, 41.6% ± 0.5 ***, 47.1% ± 0.9 ***, and 43.4% ± 1.4 *** of apoptotic cells were found to be present for free 5-ALA, 5-ALA/Au–Ag-PEG, and 5-ALA/Au–Ag-PEG-Ab NCs without laser light irradiation, respectively. These results confirm that more PpIX was produced by 5-ALA for the NCs than when compared with free 5-ALA PDT alone. The actively targeted final NC provided the highest percentage of apoptotic cell death (62.4% ± 2.8 **) in the presence of laser light irradiation. Additionally, an increase in the early and late apoptotic forms of cell death from 54.9% ± 2.7 ** for 5-ALA/Au–Ag-PEG NCs to 62.4% ± 2.8 ** for the final NC PDT treatment validated the fact that conjugation of 5-ALA to the anti HER-2 Ab actively enhanced the PS’s uptake and so improved PDT treatment outcomes.

## 4. Conclusions

A final NC consisting of alloyed bimetallic Au–Ag NPs, PEG, 5-ALA, and anti HER-2 Ab was successfully synthesized for the PDT treatment of in vitro cultured MCF-7 breast cancer cells. The conjugation of the PS and Ab took place via electrostatic interactions resulting in a final NC with a hydrodynamic size of 185.6 ± 1.4 nm. The cellular uptake of the active targeted NCs and successful internalization of the final NC within the cytoplasm of MCF-7 cancer cells were observed [13]. Furthermore, the final NC significantly enhanced the intracellular PpIX accumulation into tumor cells when compared with free 5-ALA application alone. Overall, the functionalization of the final NC with anti HER-2 Abs further increased the PS’s active subcellular localization via HER-2-receptor-mediated endocytosis and so under laser light irradiation at a wavelength of 636 nm proved to be a highly effective nanoplatform for eradicating in vitro cultured MCF-7 cells through PDT-induced favorable apoptotic forms of cell death.

## Figures and Tables

**Table 1:** Size, hydrodynamic diameter, zeta potential, and polydispersity index of Ab, 5-ALA, and nanoconjugates.

NPs/NCs	Hydrodynamic Size (nm) *DLS*	Polydispersity Index *DLS*	Zeta Potential (mV) *ELS*
Anti-HER-2 Ab	-	-	−3.6 ± 1.2
5-ALA	-	-	−1.3 ± 0.7
Citrate Au–Ag NPs	33.8 ± 4.4	0.71 ± 0.08	−21.0 ± 0.3
Au–Ag-PEG	154.0 ± 1.2	0.29 ± 0.01	7.5 ± 0.7
5-ALA/Au–Ag-PEG	192.4 ± 3.0	0.26 ± 0.04	5.5 ± 0.8
5-ALA/Au–Ag-PEG-Ab	185.6 ± 1.4	0.29 ± 0.02	5.8 ± 0.6

## Data Availability

The datasets generated during and/or analyzed during the current study are available from the corresponding author upon request.

## Abbreviations

ABC	ATP binding cassette
Abs	Antibodies
5-ALA	5-aminolevulinic acid
ATP	Adenosine triphosphate
DLS	Dynamic light scattering
DMEM	Dulbecco’s modified eagle’s medium
EDS	Energy dispersive spectroscopy
EPR	Enhanced permeability and retention
FBS	Fetal bovine serum
FECH	Ferrochelatase
ICP-MS	Inductively coupled plasma mass spectrometry
LSPR	Localized surface plasmon resonance
NC	Nanoconjugate
NPs	Nanoparticles
PBS	Phosphate-buffered saline
PDI	Polydispersity indexes
PDT	Photodynamic therapy
PEPT	Peptide transporter
PpIX	Protoporphyrin IX
PS	Photosensitizer
ROS	Reactive oxygen species
SLC	Solute carrier
TPDT	Targeted photodynamic therapy
ZP	Zeta potential

## References

[1] Crescenzi E., Varriale L., Iovino M., Chiaviello A., Veneziani B.M., Palumbo G. (2004). Photodynamic therapy with indocyanine green complements and enhances low-dose cisplatin cytotoxicity in MCF-7 breast cancer cells. Mol. Cancer Ther..

[2] Banerjee S., MacRobert A., Mosse C., Periera B., Bown S., Keshtgar M. (2017). Photodynamic therapy: Inception to application in breast cancer. Breast.

[3] Hopper C. (2000). Photodynamic therapy: A clinical reality in the treatment of cancer. Lancet Oncol..

[4] Wu J., Han H., Jin Q., Li Z., Li H., Ji J. (2017). Design and proof of programmed 5-aminolevulinic acid prodrug nanocarriers for targeted photodynamic cancer therapy. ACS Appl. Mater. Interfaces.

[5] Guo S., Sun X., Cheng J., Xu H., Dan J., Shen J., Zhou Q., Zhang Y., Meng L., Cao W. (2013). Apoptosis of THP-1 macrophages induced by protoporphyrin IX-mediated sonodynamic therapy. Int. J. Nanomed..

[6] Reinert M., Piffaretti D., Wilzbach M., Hauger C., Guckler R., Marchi F., D’angelo M.L. (2019). Quantitative modulation of PpIX fluorescence and improved glioma visualization. Front. Surg..

[7] Kennedy J., Pottier R., Pross D. (1990). Photodynamic therapy with endogenous protoporphyrin: IX: Basic principles and present clinical experience. J. Photochem. Photobiol. B Biol..

[8] Ohgari Y., Nakayasu Y., Kitajima S., Sawamoto M., Mori H., Shimokawa O., Matsui H., Taketani S. (2005). Mechanisms involved in δ-aminolevulinic acid (ALA)-induced photosensitivity of tumor cells: Relation of ferrochelatase and uptake of ALA to the accumulation of protoporphyrin. Biochem. Pharmacol..

[9] Landes R., Illanes A., Goeppner D., Gollnick H., Friebe M. (2018). A study of concentration changes of Protoporphyrin IX and Coproporphyrin III in mixed samples mimicking conditions inside cancer cells for Photodynamic Therapy. PLoS ONE.

[10] Yano S., Hirohara S., Obata M., Hagiya Y., Ogura S.-i., Ikeda A., Kataoka H., Tanaka M., Joh T. (2011). Current states and future views in photodynamic therapy. J. Photochem. Photobiol. C Photochem. Rev..

[11] Yang J., Xia Y., Liu X., Jiang S., Xiong L. (2010). Desferrioxamine shows different potentials for enhancing 5-aminolaevulinic acid-based photodynamic therapy in several cutaneous cell lines. Lasers Med. Sci..

[12] Feng Y., Liu L., Hu S., Liu Y., Ren Y., Zhang X. (2016). Förster resonance energy transfer properties of a new type of near-infrared excitation PDT photosensitizer: CuInS 2/ZnS quantum dots-5-aminolevulinic acid conjugates. RSC Adv..

[13] Danhier F., Feron O., Préat V. (2010). To exploit the tumor microenvironment: Passive and active tumor targeting of nanocarriers for anti-cancer drug delivery. J. Control. Release.

[14] Shi Y., Van der Meel R., Chen X., Lammers T. (2020). The EPR effect and beyond: Strategies to improve tumor targeting and cancer nanomedicine treatment efficacy. Theranostics.

[15] Kirtane A.R., Kalscheuer S.M., Panyam J. (2013). Exploiting nanotechnology to overcome tumor drug resistance: Challenges and opportunities. Adv. Drug Deliv. Rev..

[16] Choi K.-H., Nam K.C., Cho G., Jung J.-S., Park B.J. (2018). Enhanced photodynamic anticancer activities of multifunctional magnetic nanoparticles (Fe3O4) conjugated with chlorin e6 and folic acid in prostate and breast cancer cells. Nanomaterials.

[17] Monroe J.D., Belekov E., Er A.O., Smith M.E. (2019). Anti-cancer photodynamic therapy properties of sulphur-doped graphene quantum dot and methylene blue preparations in MCF-7 breast cancer cell culture. Photochem. Photobiol..

[18] Wang B.-Y., Liao M.-L., Hong G.-C., Chang W.-W., Chu C.-C. (2017). Near-infrared-triggered photodynamic therapy toward breast cancer cells using dendrimer-functionalized upconversion nanoparticles. Nanomaterials.

[19] Yu Z., Ge Y., Sun Q., Pan W., Wan X., Li N., Tang B. (2018). A pre-protective strategy for precise tumor targeting and efficient photodynamic therapy with a switchable DNA/upconversion nanocomposite. Chem. Sci..

[20] Yu Z., Xia Y., Xing J., Li Z., Zhen J., Jin Y., Tian Y., Liu C., Jiang Z., Li J. (2018). Y 1-receptor–ligand-functionalized ultrasmall upconversion nanoparticles for tumor-targeted trimodality imaging and photodynamic therapy with low toxicity. Nanoscale.

[21] Ramírez-García G., Panikar S.S., López-Luke T., Piazza V., Honorato-Colin M.A., Camacho-Villegas T., Hernández-Gutiérrez R., De la Rosa E. (2018). An immunoconjugated up-conversion nanocomplex for selective imaging and photodynamic therapy against HER2-positive breast cancer. Nanoscale.

[22] Feng Y., Wu Y., Zuo J., Tu L., Que I., Chang Y., Cruz L.J., Chan A., Zhang H. (2019). Assembly of upconversion nanophotosensitizer in vivo to achieve scatheless real-time imaging and selective photodynamic therapy. Biomaterials.

[23] Kim M., Lee J.H., Nam J.M. (2019). Plasmonic photothermal nanoparticles for biomedical applications. Adv. Sci..

[24] Manson J., Kumar D., Meenan B.J., Dixon D. (2011). Polyethylene glycol functionalized gold nanoparticles: The influence of capping density on stability in various media. Gold Bull..

[25] Huang X., Jain P.K., El-Sayed I.H., El-Sayed M.A. (2008). Plasmonic photothermal therapy (PPTT) using gold nanoparticles. Lasers Med. Sci..

[26] Gilroy K.D., Ruditskiy A., Peng H.-C., Qin D., Xia Y. (2016). Bimetallic nanocrystals: Syntheses, properties, and applications. Chem. Rev..

[27] Sheny D., Mathew J., Philip D. (2011). Phytosynthesis of Au, Ag and Au–Ag bimetallic nanoparticles using aqueous extract and dried leaf of Anacardium occidentale. Spectrochim. Acta A Mol. Biomol. Spectrosc..

[28] Huang X., El-Sayed I.H., Qian W., El-Sayed M.A. (2006). Cancer cell imaging and photothermal therapy in the near-infrared region by using gold nanorods. J. Am. Chem. Soc..

[29] Tripathi K., Driskell J.D. (2018). Quantifying bound and active antibodies conjugated to gold nanoparticles: A comprehensive and robust approach to evaluate immobilization chemistry. ACS Omega.

[30] Yokota S. (2010). Preparation of colloidal gold particles and conjugation to protein A, IgG, F (ab’) 2, and streptavidin. Immunoelectron Microscopy.

[31] Sokolov K., Follen M., Aaron J., Pavlova I., Malpica A., Lotan R., Richards-Kortum R. (2003). Real-time vital optical imaging of precancer using anti-epidermal growth factor receptor antibodies conjugated to gold nanoparticles. Cancer Res..

[32] Ishikawa T., Kajimoto Y., Inoue Y., Ikegami Y., Kuroiwa T. (2015). Critical role of ABCG2 in ALA-photodynamic diagnosis and therapy of human brain tumor. Adv. Cancer Res..

[33] Deng F., Sjöstedt N., Kidron H. (2016). The effect of albumin on MRP2 and BCRP in the vesicular transport assay. PLoS ONE.

[34] Sharma S., Jajoo A., Dube A. (2007). 5-Aminolevulinic acid-induced protoporphyrin-IX accumulation and associated phototoxicity in macrophages and oral cancer cell lines. J. Photochem. Photobiol. B Biol..

[35] Steinbach P., Wedmgandt H., Baumgartner R., Kriegmair M., Hofstädter F., Knüchel R. (1995). Cellular fluorescence of the endogenous photosensitizer protoporphyrin IX following exposure to 5-aminolevulinic acid. Photochem. Photobiol..

[36] Liu W., Baer M.R., Bowman M.J., Pera P., Zheng X., Morgan J., Pandey R.A., Oseroff A.R. (2007). The tyrosine kinase inhibitor imatinib mesylate enhances the efficacy of photodynamic therapy by inhibiting ABCG2. Clin. Cancer Res..

[37] An R., Hagiya Y., Tamura A., Li S., Saito H., Tokushima D., Ishikawa T. (2009). Cellular phototoxicity evoked through the inhibition of human ABC transporter ABCG2 by cyclin-dependent kinase inhibitors in vitro. Pharm. Res..

[38] Golding J., Wardhaugh T., Patrick L., Turner M., Phillips J., Bruce J., Kimani S. (2013). Targeting tumour energy metabolism potentiates the cytotoxicity of 5-aminolevulinic acid photodynamic therapy. Br. J. Cancer.

[39] Lupusoru R.V., Pricop D.A., Uritu C.M., Arvinte A., Coroaba A., Esanu I., Zaltariov M.F., Silion M., Stefanescu C., Pinteala M. (2020). Effect of TAT-DOX-PEG irradiated gold nanoparticles conjugates on human osteosarcoma cells. Sci. Rep..

[40] Choi K.Y., Min K.H., Yoon H.Y., Kim K., Park J.H., Kwon I.C., Choi K., Jeong S.Y. (2011). PEGylation of hyaluronic acid nanoparticles improves tumor targetability in vivo. Biomaterials.

[41] de Oliveira Gonçalves K., da Silva M.N., Sicchieri L.B., de Oliveira Silva F.R., de Matos R.A., Courrol L.C. (2015). Aminolevulinic acid with gold nanoparticles: A novel theranostic agent for atherosclerosis. Analyst.

[42] Aishwarya S., Sanjay K. (2018). Conjugation study of 5-aminolevulinic acid with microbial synthesized gold nanoparticles to evaluate its effect on skin melanoma and epidermoid carcinoma cell lines using photodynamic cancer therapy. Gold Bull..

[43] Chung C.-W., Chung K.-D., Jeong Y.-I., Kang D.H. (2013). 5-aminolevulinic acid-incorporated nanoparticles of methoxy poly (ethylene glycol)-chitosan copolymer for photodynamic therapy. Int. J. Nanomed..

[44] Adegoke O., Morita M., Kato T., Ito M., Suzuki T., Park E.Y. (2017). Localized surface plasmon resonance-mediated fluorescence signals in plasmonic nanoparticle-quantum dot hybrids for ultrasensitive Zika virus RNA detection via hairpin hybridization assays. Biosens. Bioelectron..

[45] Xu H., Liu C., Mei J., Yao C., Wang S., Wang J., Li Z., Zhang Z. (2012). Effects of light irradiation upon photodynamic therapy based on 5-aminolevulinic acid–gold nanoparticle conjugates in K562 cells via singlet oxygen generation. Int. J. Nanomed..

[46] Mulvaney P., Klabunde K.J. (2001). Metal nanoparticles: Double layers, optical properties, and electrochemistry. Nanoscale Materials in Chemistry.

[47] Kuipers B.J., Gruppen H. (2007). Prediction of molar extinction coefficients of proteins and peptides using UV absorption of the constituent amino acids at 214 nm to enable quantitative reverse phase high-performance liquid chromatography–mass spectrometry analysis. J. Agric. Food Chem..

[48] Zhao X., Yang C.-X., Chen L.-G., Yan X.-P. (2017). Dual-stimuli responsive and reversibly activatable theranostic nanoprobe for precision tumor-targeting and fluorescence-guided photothermal therapy. Nat. Commun..

[49] Davatgaran Taghipour Y., Kharrazi S., Amini S.M. (2018). Antibody conjugated gold nanoparticles for detection of small amounts of antigen based on surface plasmon resonance (SPR) spectra. Nanomed. Res. J..

[50] Yao G.-Y., Liu Q.-L., Zhao Z.-Y. (2018). Studied localized surface plasmon resonance effects of Au nanoparticles on TiO_2_ by FDTD simulations. Catalysts.

[51] Koizumi N., Hanai T. (1964). Dielectric properties of polyethylene glycols: Dielectric relaxation in solid state (special issue on polymer chemistry, I). Bull. Inst. Chem. Res. Kyoto Univ..

[52] Petryayeva E., Krull U.J. (2011). Localized surface plasmon resonance: Nanostructures, bioassays and biosensing—A review. Anal. Chim. Acta.

[53] Khaing Oo M.K., Yang X., Du H., Wang H. (2008). 5-aminolevulinic acid-conjugated gold nanoparticles for photodynamic therapy of cancer. Nanomedicine.

[54] Yoo C.Y., Seong J.S., Park S.N. (2016). Preparation of novel capsosome with liposomal core by layer-by-Layer self-assembly of sodium hyaluronate and chitosan. Colloids Surf. B.

[55] de Oliveira Gonçalves K., Vieira D.P., Levy D., Bydlowski S.P., Courrol L.C. (2020). Uptake of silver, gold, and hybrids silver-iron, gold-iron and silver-gold aminolevulinic acid nanoparticles by MCF-7 breast cancer cells. Photodiagn. Photodyn. Ther..

[56] Shaker M.N., Ramadan H.S., Mohamed M.M., Roston G.D. (2014). Enhanced photodynamic efficacy of PLGA-encapsulated 5-ALA nanoparticles in mice bearing Ehrlich ascites carcinoma. Appl. Nanosci..

[57] Punjabi A., Wu X., Tokatli-Apollon A., El-Rifai M., Lee H., Zhang Y., Wang C., Liu Z., Chan E.M., Duan C. (2014). Amplifying the red-emission of upconverting nanoparticles for biocompatible clinically used prodrug-induced photodynamic therapy. ACS Nano.

[58] Aghamiri S., Jafarpour A., Shoja M. (2019). Effects of silver nanoparticles coated with anti-Her2 on irradiation efficiency of Skbr3 breast cancer cells. IET Nanobiotechnol..

[59] Juluri B.K., Zheng Y.B., Ahmed D., Jensen L., Huang T.J. (2008). Effects of geometry and composition on charge-induced plasmonic shifts in gold nanoparticles. J. Phys. Chem. C.

[60] Hoener B.S., Zhang H., Heiderscheit T.S., Kirchner S.R., De Silva Indrasekara A.S., Baiyasi R., Cai Y., Nordlander P., Link S., Landes C.F. (2017). Spectral response of plasmonic gold nanoparticles to capacitive charging: Morphology effects. J. Phys. Chem. Lett..

[61] Gadmar Ø.B., Moan J., Scheie E., Ma L.-W., Peng Q. (2002). The stability of 5-aminolevulinic acid in solution. J. Photochem. Photobiol. B Biol..

[62] Steluti R., De Rosa F.S., Collett J., Tedesco A.C., Bentley M.V.L.B. (2005). Topical glycerol monooleate/propylene glycol formulations enhance 5-aminolevulinic acid in vitro skin delivery and in vivo protophorphyrin IX accumulation in hairless mouse skin. Eur. J. Pharm. Biopharm..

[63] Wang Y., Yang M., Qian J., Xu W., Wang J., Hou G., Ji L., Suo A. (2019). Sequentially self-assembled polysaccharide-based nanocomplexes for combined chemotherapy and photodynamic therapy of breast cancer. Carbohydr. Polym..

[64] Melamed J.R., Edelstein R.S., Day E.S. (2015). Elucidating the fundamental mechanisms of cell death triggered by photothermal therapy. ACS Nano.

